# Knowledge, attitudes, and practice gap in female condoms utilization among future health professionals. A cross-sectional study

**DOI:** 10.3389/frph.2026.1851756

**Published:** 2026-06-18

**Authors:** William Willington Kwashiga Awuku, Micheal Agbesi Lawer, Geoffina Esi Armoh, Francisca Kafui Agbenya, Suuk Seidu, Joshua Oppong, Felix Nyande, Hubert Amu

**Affiliations:** 1 Department of Nursing, School of Nursing and Midwifery, University of Health and Allied Sciences, Ho, Ghana; 2 Department of Population and Behavioural Sciences, Fred N. Binka School of Public Health, University of Health and Allied Sciences, Hohoe, Ghana

**Keywords:** attitude, contraceptives, female condom, Ghana, knowledge, nursing student, sexual and reproductive health, utilization

## Abstract

**Background:**

Nursing student as a future frontline healthcare professionals play a critical role in promoting sexual and Reproductive Health (SRH) and influencing contraceptive behaviours within communities. However, there is limited evidence on their knowledge, attitude and utilization of female condoms among these health trainees. We, therefore, examined the knowledge, attitude, and utilization of female condoms among nursing students at the Nursing Training College, Ho.

**Methods:**

A descriptive cross-sectional study was conducted among 402 female students selected through simple random sampling at the NTC-Ho. A structured questionnaire was used to collect data and analyzed using Stata Version 17. Chi-square tests and logistic regression were performed to identify predictors of female condom utilization, with a *p*-value <0.05 considered statistically significant for all analysis.

**Results:**

We found that more than half (55.5%) had poor knowledge with regard to the female condom. More than half (57.7%) had a positive attitude; however, utilization was low (7.7%). Perceived challenges for utilization were difficulty in access (25.7%) and preference for other contraceptives (21.8%), especially male condoms (47.8%). Marital status emerged as a significant predictor of female condom use (OR = 4.31, 95% CI: 1.10–16.82, *p* = 0.036).

**Conclusion:**

Our findings revealed that while participants' positive attitude was high, actual utilization remained very low. Integrating hands-on demonstrations of female condom, enhanced availability, incorporation of partner communication and negotiation skills training, will be essential to improve uptake and contribute to the achievement of Sustainable Development Goal (SDG) target 3.7, which aims to reduce unintended pregnancies and sexually transmitted infections, including HIV by 2030.

## Background

Sexual and Reproductive Health (SRH) remains a major global public health concern, particularly among adolescents and young adults, who are disproportionately affected by sexually transmitted infections (STIs), unintended pregnancies, and maternal mortality (1). Globally, an estimated 374 million new cases of curable STIs occur annually among individuals aged 15–49 years, while nearly 46% of the approximately 208 million pregnancies recorded each year are unintended, contributing significantly to maternal morbidity and mortality in low- and middle-income countries (LMIC) (2, 3). In response to addressing the issue of unintended pregnancy and prevention of STIs, Sustainable Development Goal (SDG) target 3.7 emphasizes universal access to sexual and reproductive health services, including family planning information and education (4).

Among available contraceptive methods, the female condom stands out as the only female-initiated method providing dual protection against both unintended pregnancy and STIs, including HIV, and it represents a female-initiated method that enhances women's autonomy over sexual health decisions (5, 6). Despite these advantages, the global utilization of female condoms remains extremely low, averaging less than 1% across most countries, particularly when compared with other modern contraceptive methods (7, 8). Several barriers contribute to the persistent low utilization. At the individual level, factors such as limited knowledge, lack of practical skills, and discomfort with insertion discourage use (9–11). Interpersonal barriers, including partner resistance, limited negotiation power, and male preference for alternative contraceptive methods, further influence uptake (10, 12). In addition, structural barriers such as poor availability, high cost, inadequate promotion within health facilities, and limited integration of female condom education into formal curricula continue to hinder utilization (9, 13). Although higher acceptance has been reported in some high-income settings with empowerment-focused SRH programs but utilization remains limited in sub-Saharan Africa (SSA) due to sociocultural norms, misinformation, partner resistance, and inconsistent availability (14).

The challenge of female condom underutilisation is particularly pronounced in SSA. SSA bears a disproportionate burden of SRH challenges, with adolescent girls and young women facing elevated risks of HIV, STIs, and unintended pregnancies. Studies in countries such as Nigeria, Kenya, and Cameroon report that fewer than 20% of sexually active women have ever used a female condom, despite general awareness of the method (14–16). These trends are similar in Ghana, with persistent adolescent pregnancy rates and an adult HIV prevalence of approximately 1.7% (17). Although female condoms were introduced in Ghana in 2,000 as part of the National Reproductive Health Policy, utilization remains poor. Reports from the Ghana Health Service indicate low patronage, particularly among young women (5).

Evidence from Ghana suggests that while awareness of female condoms may be relatively high, detailed knowledge and actual utilization remain limited due to poor access, inadequate practical education, and negative or ambivalent partner dynamics (5, 18, 19). Nursing students occupy a uniquely important position in the SRH landscape, as young women who face the same reproductive health vulnerabilities as their peers, and as future frontline healthcare providers, who will counsel patients on contraceptive choices. Their knowledge, attitudes, and personal practices regarding female condoms will directly influence the quality of information and services they provide to patients. Despite this, existing studies in Ghana have largely focused on male condoms and hormonal contraceptives (20–22), while a study on female condom use primarily targeted the general population (5) with limited attention to female condom use among nursing students who are future frontline health professionals. Therefore, this study assessed the knowledge, attitudes, and utilization of female condoms among female nursing students at NTC-Ho.

## Methods and materials

Our study adopts the STROBE guidelines for a quantitative study (23).

### Study setting

**Figure 1:**
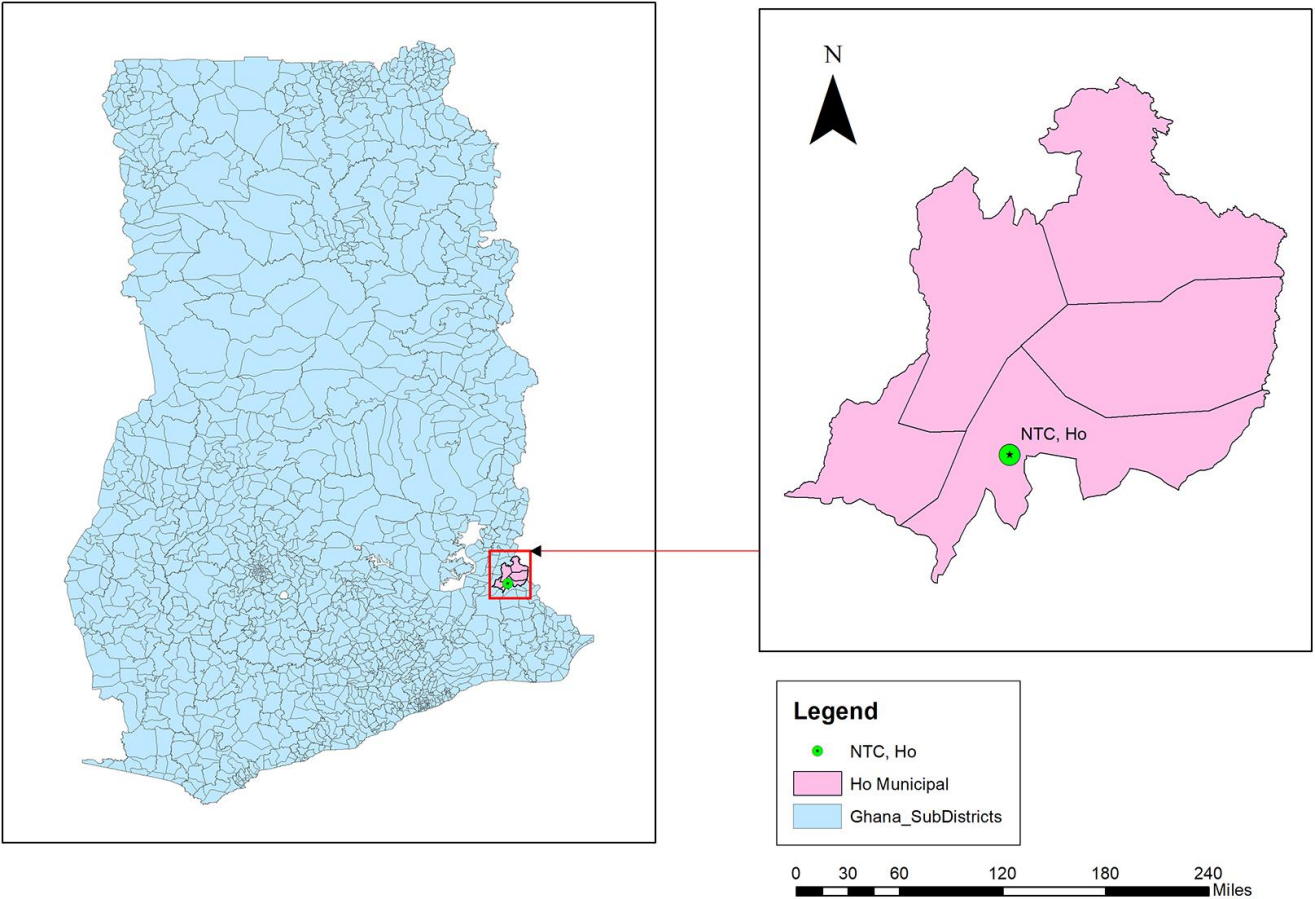
Map showing the location of Ho municipal in the volta region of Ghana. Source: Author-generated.

Our study was conducted at Nurses Training College Ho, located in Ho Municipality in the Volta Region of Ghana, as shown in Figure 1. The college is a public nursing training institution under the Ministry of Health and regulated by the Nursing and Midwifery Council of Ghana, with a mandate to train professional nurses (24). The institution admits students from across the Volta Region and other parts of Ghana and offers diploma nursing programmes that include sexual and reproductive health training. The college was selected because it trains future frontline healthcare providers and is situated in a region where female condom utilization remains low.

### Study design and population

Our study adopted a descriptive cross-sectional design to assess the knowledge, Attitude and utilization of female condoms among nursing students at Ho.

Our study was conducted among female students in HNTC and excluded all female students who were not available at the time of data collection.

### Sample size and procedure

Our sample size was determined using the Yamane formula (25) ($n=\frac{N}{1+N{\left(e\right)}^{2}}$, *n* = sample size, *N* = total population size (1,171), and *e* = margin of error (0.05 for 95% confidence). With 10% non-response rate. The minimum sample required for the study was 331. However, 402 participated in the study to improve the statistical power and representativeness of our study. Participants were recruited from 06/11/2025 to 28/11/2025 for data collection. Proportionate allocation was done based on the levels (Level 100, 200, 300).

### Sampling

Our study adopted a simple random sampling method to select participants. At each level, a list of all female nursing students was obtained from the college administration and served as the sampling frame. Each student was assigned a unique identification number, and participants were selected using a computer-generated random number sequence until the required sample size for each level was achieved. In cases where a selected participant was unavailable or declined participation, replacement was done using the next pre-generated random number on the list to maintain the randomness of the sampling procedure.

### Data collection instrument and procedure

A structured questionnaire deployed via Google forms were used to collect data. The questionnaire was structured into four sections. Section A comprised the demographic characteristics of the respondents (age, marital status, ethnicity, academic level, programme and sexually active). Section B consisted of the respondents' knowledge of the FC (the purpose of the female condom, the material used, effectiveness with correct use, and time of insertion before sexual intercourse). Section C: Attitude towards Female Condom. Section D comprised the utilization of female condom (ever used, first time use, reasons for not using and preferred contraceptive). The knowledge items assessed distinct factual domains rather than a single underlying construct; therefore, internal consistency reliability testing was not performed. Similarly, the attitude items assessed different perceptions toward female condom use, including cultural acceptability, empowerment, embarrassment, perceived effectiveness, professional advocacy, and intention to use. Due to the multidimensional nature of these items, they were primarily analyzed as descriptive indicators of attitudes toward female condoms rather than as a unidimensional psychometric scale.

Data were collected face-to-face by five members of the team who were final year undergraduate nursing students at the time of the data collection. The research assistants were trained on the objectives of the study, ethical considerations, and standardized administration of the questionnaire to minimize interviewer bias and ensure consistency in data collection procedures. Data were collected during break sessions at the school premises.

### Study variables

The primary outcome variable was utilization of the female condom. knowledge, attitude toward female condoms, and socio-demographic (age, sex, marital status, ethnicity, religion, academic year, program, sexually active) were treated as explanatory variables.

Utilization was assessed using a single item asking whether the participant had ever used a female condom. Participants who responded “Yes” were categorized as “Ever used”, while those who responded “No” were categorized as “Never used”.

Knowledge was assessed using four items covering the purpose of the female condom, the material used, effectiveness with correct use, and time of insertion before sexual intercourse. Each item was further dichotomized into “know” and “don't know” based on the responses. Participants who provided a correct response for an item were scored as 1 (“know”), while incorrect responses were scored 0 (“don't know”). A composite knowledge score was generated by summing all items with a total score of 4. Knowledge was dichotomised based on scores, with those below 3 categorised as “0” Poor and equal to or above 3, “1” Good knowledge. A cut-off score of 3 out of 4 (75%) was adopted because nursing students are expected to demonstrate a relatively high level of knowledge regarding female condom.

Attitude was assessed using six items measured on a 5-point Likert scale ranging from strongly agree to strongly disagree. Four items were positively worded, while two were negatively worded (Item 1 and Item 3). To ensure consistency in the direction of scoring, the negatively worded items were reverse-coded during data analysis. Each item was scored from 1 to 5, giving a total possible score of 30. A composite attitude variable was generated and dichotomized into positive and negative attitudes with a score <20 “0” Negative, ≥20 “1” Positive. The cut-off score of 20 was used because it represents responses above the neutral midpoint of the scale. This indicates a generally favourable attitude towards female condom use among nursing students.

### Data analysis

Data were analysed using STATA v17.0. Descriptive statistics, such as frequencies and percentages, were performed on all variables (socio-demographic characteristics, knowledge, attitude, utilization). Inferential statistics (Chi-square and logistic regressions) were also performed to determine the factors associated with utilization. A *p* < 0.05 was considered statistically significant at a 95% confidence interval. Model assumptions for logistic regression were considered before analysis. Independence of observations was ensured through the study design, as each participant contributed only one response. Since crude logistic regression models were fitted separately for each predictor and all explanatory variables were categorical, assumptions related to multicollinearity and linearity were not applicable.

### Ethical considerations

Ethical approval for our study was obtained from the University of Health and Allied Sciences Research Ethics Committee (UHAS-REC) [Reference number: UHAS-REC A.10(18) 24–25]. Permission to conduct the study was also granted by the authorities of Ho Nursing Training College. All participants were informed about the purpose, procedures, potential benefits, and minimal risks of the study before participation. Written consent was obtained from each participant before participation. Participation was entirely voluntary, and respondents were informed of their right to decline participation or withdraw from the study at any time without any academic or personal consequences. To ensure confidentiality and anonymity, no personal identifiers were collected. Completed questionnaires were securely stored, and electronic data were password-protected and accessible only to the research team. Data were used strictly for research purposes. Our study adhered to the ethical principles outlined in the Declaration of Helsinki for research involving human participants, including respect for persons, beneficence, and justice.

## Results

### Socio-demographic characteristics

Table 1 presents the socio-demographic characteristics of female nursing students. The majority (68.7%) of participants were aged between 20 and 24 years, and almost all of them were never married (97.0%). Most were of Ewe (74.4%) and the majority were Christians (95.0%). In terms of academic level, the largest group was first-year students (68.2%), and by program, the majority were enrolled in Registered General Nursing (44.5%). Regarding sexual activity, most participants (61.0%) reported not being sexually active in the past seven months.

**Table 1:** Socio-demographic characteristics.

Variable	Freq (*N*)	Percent (%)
Age		
<20	74	18.4
20–24	276	68.7
25–29	41	10.2
30+	11	2.7
Marital Status		
Single	390	97.0
Married	12	3.0
Ethnicity		
Akan	44	10.9
Ewe	299	74.4
Ga-Dangme	34	8.5
Other	25	6.2
Religion		
Christianity	382	95.0
Islam	20	5.0
Academic level		
First year	274	68.2
Second year	85	21.1
Third year	43	10.7
Programme		
Registered community nursing (RCN)	87	21.6
Registered general nursing (RGN)	179	44.6
Registered nurse assistant	136	33.8
Sexually active (past 7 months)		
No	245	61.0
Yes	157	39.0

### Knowledge of female condom

Table 2 presents the knowledge on female condoms among the respondents. About the purpose of the female condom, the majority of respondents (85.3%) correctly indicated that it is used to prevent both pregnancy and sexually transmitted infections, including HIV. Concerning the material used to manufacture female condoms, more than half (60.7%) indicated that they did not know the material used. Most respondents (65.7%) correctly reported that female condoms are most effective when used correctly and consistently. However, regarding the timing of insertion, a high majority of respondents (70.2%) indicated that they did not know that female condoms can be inserted up to 8 h before sexual intercourse.

**Table 2:** Knowledge on female condom.

Variable	Freq	Percent (%)
Purpose of Female Condom (*N* = 402)		
Prevent both pregnancy and STIs/HIV	343	85.3
Prevent STIs/HIV only	5	1.3
Prevent pregnancy only	7	1.7
I don't know	47	11.7
Material Used for Female Condom (*N* = 402)		
Latex	80	19.9
Leather	1	0.2
Nitrile	18	4.5
Polyurethane	59	14.7
I don't know	244	60.7
Female condoms are most effective when (*N* = 402)		
Used correctly & consistently	264	65.7
Used with male condom simultaneously	60	14.9
All of the above	78	19.4
Insert up to 8 h before sex (*N* = 402)		
True	120	29.9
False	282	70.1
Source of information* (*n* = 340)		
Public health campaigns	65	19.1
College curriculum/lectures	60	17.6
Friends/peers	70	20.6
Social media/radio/TV	55	16.2
Health workers/clinics	50	14.7
Never learned about them	40	11.8

* Multiple responses.

### Attitude towards female condom use

Table 3 presents attitudes toward female condoms among respondents. The majority of participants agreed that female condoms are reliable for preventing STIs and pregnancy (52.7%) and that nursing students should advocate for female condom use (56.2%). Most also agreed that female condoms give women more control over their sexual health (33.1%). Regarding cultural or religious norms making it difficult to use female condoms (7.7%), strongly agree and the likelihood of using a female condom if available (29.6%) responded neutral. Participants mostly disagreed with feeling embarrassed to suggest using a female condom (35.8%).

**Table 3:** Attitude towards female condom use.

Statement	Strongly agree *n* (%)	Agree *n* (%)	Neutral *n* (%)	Disagree *n* (%)	Strongly disagree *n* (%)
Cultural or religious norms make it difficult to use female condoms	31 (7.7)	98 (24.4)	115 (28.6)	97 (24.1)	61 (15.2)
Female condoms give women more control over their sexual health	43 (10.7)	133 (33.1)	116 (28.9)	74 (18.4)	36 (8.9)
I would feel embarrassed to suggest using a female condom	18 (4.5)	57 (14.2)	98 (24.4)	144 (35.8)	85 (21.1)
Female condoms are reliable for preventing STIs and pregnancy	107 (26.6)	212 (52.7)	46 (11.4)	20 (5.0)	17 (4.3)
Nursing students should advocate for female condom use	87 (21.6)	226 (56.2)	62 (15.4)	12 (3.0)	15 (3.7)
If available, how likely would you be to use a female condom	47 (11.7)	91 (22.6)	119 (29.6)	84 (20.9)	61 (15.2)

Table 4 presents other variables on the utilization of female condoms among the participants. The majority of (77.4%) reported using it within the last 0–3 months. Regarding discussions with healthcare providers, 81.1% of participants had never talked to a provider about female condoms. Among reasons for not using female condoms, the most commonly reported factor was difficult to access (19.5%). In terms of the source of female condom access, the majority of participants, 75.4%, reported not knowing where their last assessment took place.

**Table 4:** Utilization of female condom.

Variable	Freq	Percent (%)
First time used female condom (*n* = 31)		
0–3 months ago	24	77.4
4–9 months ago	4	12.9
10–12 months ago	3	9.7
Discussed female condom with healthcare provider (*n* = 402)		
Yes	76	18.9
No	326	81.1
Reason for not using* (*n* = 518)		
Uncomfortable to use	118	22.8
Difficult to access	133	25.7
Prefer other contraceptives	113	21.8
Never heard of them	121	23.4
Partner refuses	33	6.4
Source of female condom access* (*n* = 435)		
Don't know	328	75.4
Local pharmacy	31	7.1
Government health facility	26	6.0
College clinic	26	6.0
Friends	24	5.5

* Multiple Responses.

**Figure 2:**
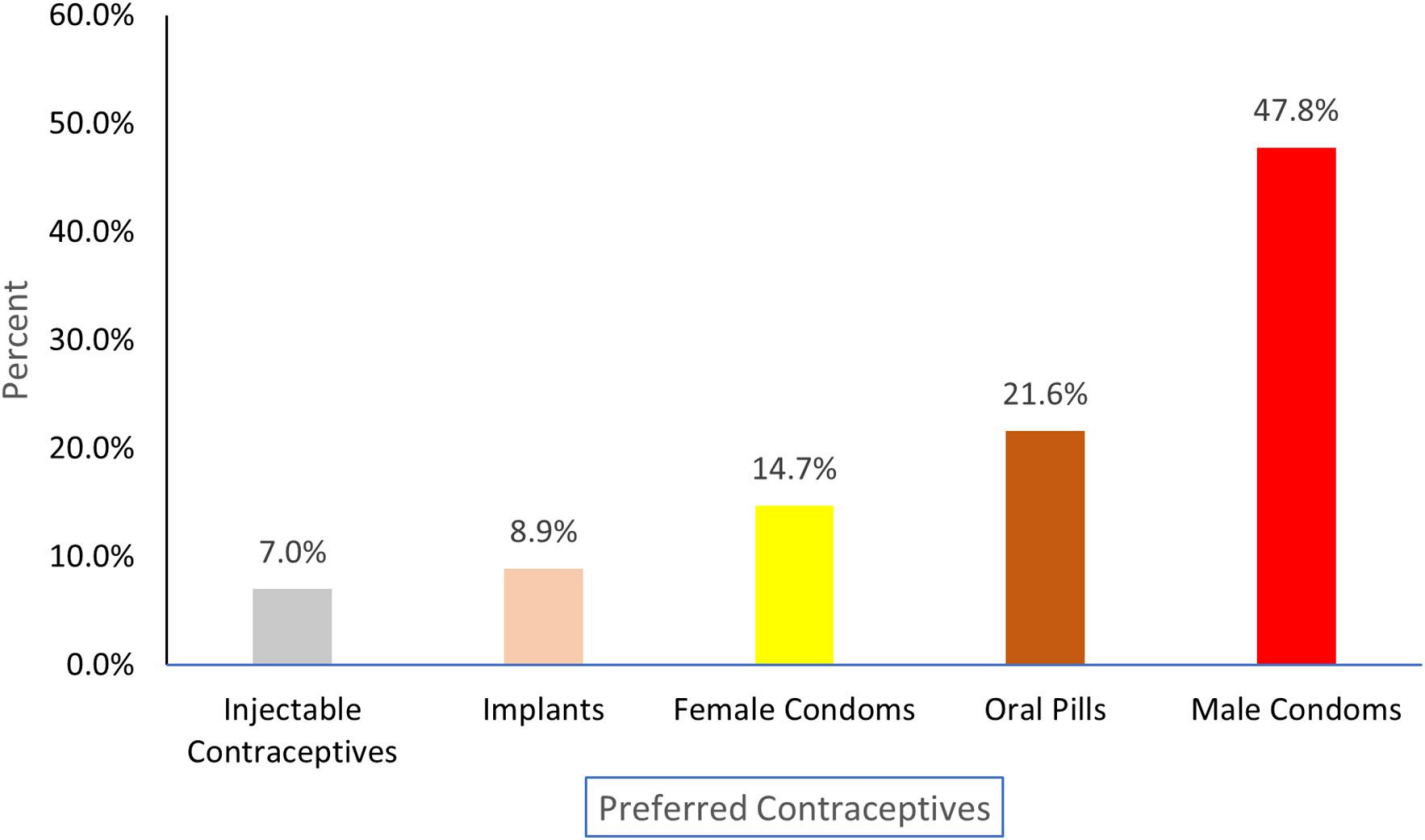
Preferred contraceptive methods among female nursing students (*N* = 402).

Figure 2 presents their preferred contraceptives. Most of them (47.8%) preferred male condoms, followed by oral pills (21.6%), with injectable contraceptives being the least (7.0).

**Figure 3:**
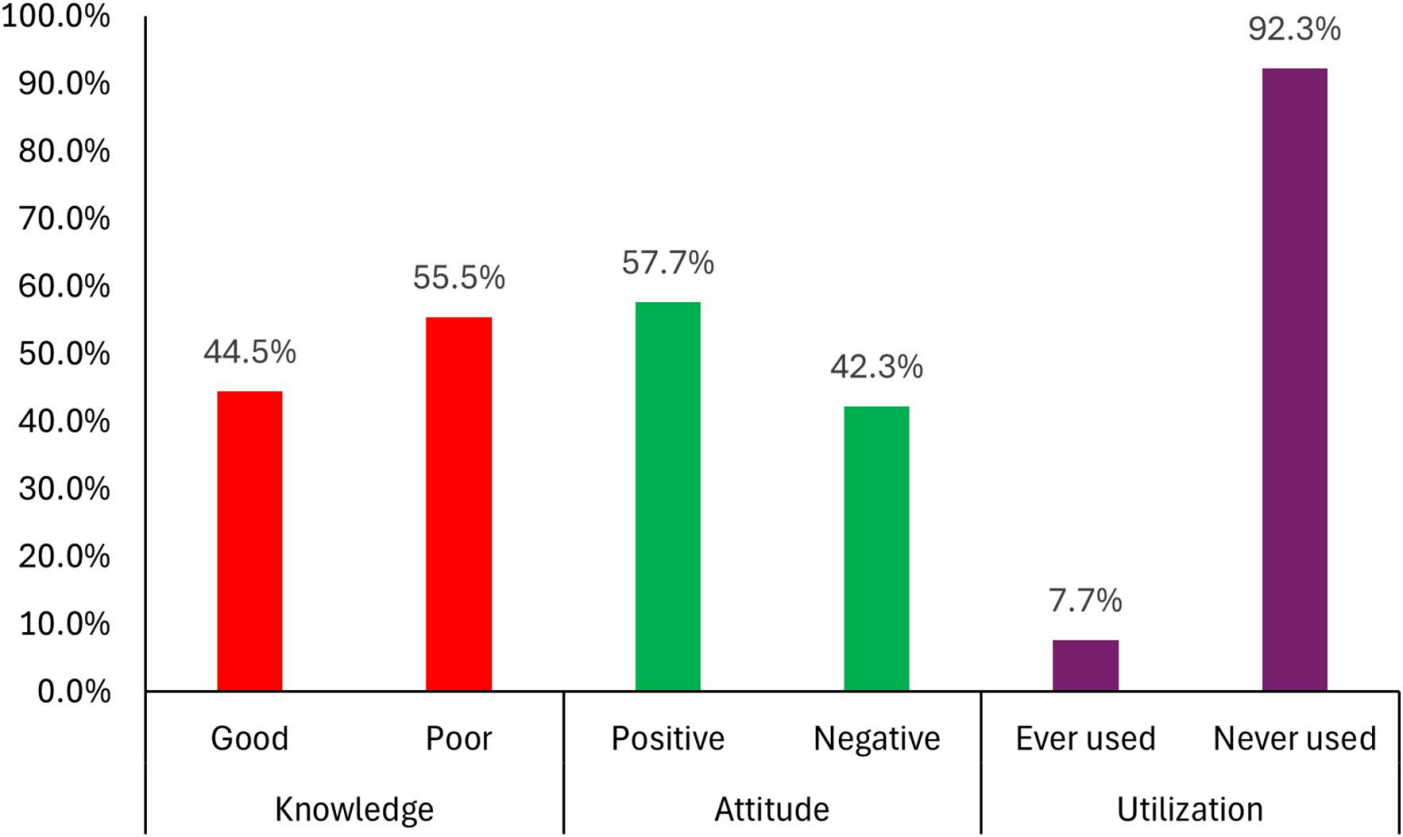
Knowledge, attitude, and utilization of female condom use among respondents.

Figure 3 presents the overall knowledge, attitude, and utilization of female condom use among respondents. More than half (55.5%) demonstrated poor knowledge of the female condom. However, a majority (57.7%) exhibited a positive attitude toward its use, while actual utilization remained low at 7.7%.

### Predictors of female condom utilization

Table 5 presents the predictors associated with the utilization of female condoms among the participants. In the bivariate analysis, marital status was significantly associated with the utilization of female condoms. Married participants were 4.3 times more likely to have used female condoms compared to those who were never married (OR = 4.31, 95% CI: 1.10–16.82, *p* = 0.036).

**Table 5:** Predictors of female condom utilization.

Variable	Female condom utilization				
	Ever used *n* (%)	Never used *n* (%)	*X* ^2^	*p*-value/f exact	OR (95% CI), *p*-value
Age group			0.849	0.838	
<20	4 (12.9)	70 (18.9)			Ref
20–24	22 (71.0)	254 (68.5)			1.52 (0.51–4.54), 0.458
25–29	4 (12.9)	37 (10.0)			1.89 (0.45–8.00), 0.386
30+	1 (3.2)	10 (2.6)			1.75 (0.18–17.27), 0.632
Marital status			5.195	0.023	
Never married	28 (90.3)	362 (97.6)			Ref
Married	3 (9.7)	9 (2.4)			4.31 (1.10–16.82), 0.036
Ethnicity			1.104	0.776	
Akan	4 (12.9)	40 (10.8)			Ref
Ewe	21 (67.7)	278 (74.9)			0.76 (0.25–2.31), 0.623
Ga-Dangme	4 (12.9)	30 (8.1)			1.33 (0.31–5.77), 0.700
Other	2 (6.5)	23 (6.2)			0.87 (0.15–5.12), 0.877
Religion			0.155	0.694	
Christianity	29 (93.6)	353 (95.2)			Ref
Islam	2 (6.4)	18 (4.8)			1.35 (0.30–6.12), 0.695
Academic year			2.045	0.361	
First year	22 (71.0)	252 (67.9)			Ref
Second year	4 (12.9)	81 (21.8)			0.57 (0.19–1.69), 0.308
Third year	5 (16.1)	38 (10.3)			1.51 (0.54–4.22), 0.435
Program			1.840	0.399	
RCN	4 (12.9)	83 (22.4)			Ref
RGN	14 (45.2)	165 (44.5)			1.76 (0.56–5.52), 0.332
Registered nurse assistant	13 (41.9)	123 (33.1)			2.19 (0.69–6.96), 0.183
Sexually active			0.002	0.967	
No	19 (61.3)	226 (60.9)			Ref
Yes	12 (38.7)	145 (39.1)			0.98 (0.46–2.09), 0.967
Attitude			1.197	0.274	
Negative	16 (51.6)	154 (41.5)			Ref
Positive	15 (48.4)	217 (58.5)			0.67 (0.32–1.39), 0.277
Knowledge level			0.091	0.762	
Poor knowledge	18 (58.1)	205 (55.3)			Ref
Good knowledge	13 (41.9)	166 (44.7)			0.89 (0.42–1.87), 0.763

## Discussion

This was a quantitative study that examined knowledge, attitudes, utilization, and factors associated with the use of female condoms among female nursing students. The findings showed that more than half of the respondents had poor knowledge (55.5%) and a positive attitude (57.7%) towards female condoms. However, utilization was very low, with only 7.7% ever using a female condom. Marital status was the only significant predictor of utilization and the majority of the female nurses preferred male condoms.

Our findings on knowledge show that almost 6 out of every ten female nursing students have poor knowledge. Many could identify the general purposes of the female condom, but knowledge about practical use was low. These findings are consistent with studies in sub-Saharan Africa and other low- and middle-income countries, where knowledge about female condoms is often limited despite some levels of reported awareness (5, 26). For example, a study by Ananga et al. reported a generally low level of awareness and knowledge of female condoms, with many women unaware of critical information about the method, its correct use, and its benefits (5). Similarly, a study in Kisumu, Kenya, reported that despite many women having heard of female condoms, a significant number had poor knowledge about them at all, highlighting persistent gaps in women's understanding of this contraceptive option (26).

Our findings that knowledge was poor may be due to several factors. Students may receive general sexual reproductive health information, but female condoms are often less emphasized than male condoms in public health curricula and health promotion messaging, with Ananga et al. suggesting that many women and young people know about female condoms in name only, without clear instruction on use (5). Again, it can be due to limited exposure of female condom. Many women and students have never seen a female condom package or been shown how to insert one correctly, limiting their detailed understanding (16). Also, cultural and social communication barriers as reported by (27). In many SSA societies, open discussion about sexual health is constrained by cultural and religious norms, leading young women to rely on peers or informal sources for information, which may be incomplete or inaccurate.

This implies that female condoms are one of the few female-initiated methods that protect against both unintended pregnancy and sexually transmitted infections, including HIV. Lack of detailed understanding reduces the likelihood that women will consider or correctly use female condoms, thereby limiting their reproductive autonomy and increasing vulnerability to poor sexual health outcomes. The findings suggest the need to strengthen educational interventions within nursing programs and the community. This includes increasing awareness of female condoms through offering practical instruction, demonstrations, and integration into routine sexual and reproductive health counseling.

Our finding on attitude is consistent with previous studies showing positive attitudes toward female condoms among young women. For instance, a study among female university students in South Africa found that students valued the dual protection female condoms offer against both pregnancy and STIs and sometimes preferred them over hormonal methods due to this advantage (28). Similarly, a study in Tanzania revealed that positive perceptions were associated with greater reported intention to use female condoms, although knowledge and use remained low overall (11). However, a study in Ghana reported mixed attitudes, with some women expressing negative views due to discomfort, perceived inconvenience, or stigma associated with female condom use (5).

Several factors may explain the positive attitudes observed among nursing students in this study. The health education background likely enhances awareness and understanding of sexual and reproductive health, even though we found that knowledge was poor. Female condoms are female-initiated methods, providing women with autonomy and control over sexual health decisions, which has been shown to positively influence attitudes (29). Nevertheless, neutral responses regarding the likelihood of use indicate that a positive attitude alone may not always translate into utilization. Positive attitudes among nursing students provide a strong foundation for peer advocacy and health promotion, as they are future health professionals who can influence community perceptions and practices.

However, the discrepancy between positive attitudes and low utilization observed warrants critical reflection. The attitude-utilization gap may reflect the social desirability bias, which may have inflated reported positive attitudes, particularly among nursing students who are trained to promote health behaviors. Second, positive attitudes may be necessary but insufficient for behavior change without addressing structural barriers such as access, affordability, and partner dynamics. The Theory of Planned Behavior suggests that intention to use a contraceptive method is influenced not only by attitudes but also by subjective norms and perceived behavioral control (30). In our study, even when nursing students hold positive attitudes, their actual use may be constrained by the limited availability of female condoms in health facilities, lack of practical skills in insertion and use, and inability to negotiate use with partners.

The low utilization is consistent with other studies from similar settings, particularly in sub-Saharan Africa (10, 31). For example, a study in Ethiopia among female commercial sex workers reported low utilization of female condoms, with barriers including limited availability, high cost, and structural factors such as inability to negotiate safer sex practices with partners (32). Similarly, among Nigerian university students, only about 8.9% had ever used them, with barriers such as unavailability, cost, and difficulty with insertion (33). While among street youth, only 4.3% have actually used a female condom (34).

However, in Uganda, the prevalence of female condom use was only 34%, which was still considered low and far below optimal levels for dual protection against unintended pregnancy and sexually transmitted infections (35). Moreover, a study in East Africa reported that although a very high proportion of respondents had heard of female condoms, with only about 22.1% reporting ever using a female condom and an even smaller proportion using one at their last sexual intercourse (36).

This finding may be due to the limited access and availability of female condoms in the institution. Accessibility and availability significantly reduce the likelihood of use; many users simply cannot easily access female condoms when needed, even when they are aware of them which was similarly reported in Shallie et al., (15). This could also be due to social and interpersonal barriers, including partner refusal, which further impede utilization. Ananga et al. reported that male partners preferred male condoms or resisted the use of female condoms, undermining women's ability to use the method even when they wanted to (5).

Female condoms are one of the few contraceptive methods that empower women to initiate protection independently and provide dual protection against unintended pregnancy and STIs, including HIV. Low utilization implies that many women, particularly young, unmarried women, are missing out on an effective tool for sexual and reproductive health. Due to the continued burden of HIV and unintended pregnancy in many LMICs, bridging the gap between awareness and utilization is key in women's reproductive health. The low uptake also suggests that knowledge alone is insufficient; therefore, interventions should focus on access, skills building, and partner communication to make female condoms a viable option for women. Improved distribution systems, subsidized pricing, enhanced social marketing, and integrated reproductive health services could help increase utilization.

Marital status was the only significant predictor for female condom use, with married participants more likely to have used a female condom than never-married peers. This finding is consistent with Shannon et al., who reported marital status as a significant predictor in Uganda (35). Similarly, Schuyler et al. reported that partner support is a central influencing factor for utilization (10). In Ghana, women reported higher preference or utilization when partners were supportive, underlining that male partner acceptance is a key social determinant of female condom use (5).

This finding could be due to the ability of women to discuss with their partners. A study indicates that women who feel confident negotiating condom use with partners and who receive partner support are more likely to use female condoms (10). At the interpersonal level, male partner attitudes toward female condoms strongly influence uptake with Mantell et al. reporting that believing that one's partner viewed female condoms positively was associated with a greater likelihood of future use, and discussing condom use with partners increased sustained use over time (37).

This implies that interventions aiming to increase female condom use must go beyond increasing awareness and positive attitudes, but rather address the skills and empowerment needed for negotiation, especially with male partners. Programs that enhance self-efficacy, negotiation skills, and insertion confidence, as well as those that engage men as supportive partners, have shown promise in improving uptake. Additionally, health system strategies that improve availability, affordability, and visibility of female condoms at multiple points of care, alongside community campaigns that challenge stigmatizing norms, are essential to converting favorable attitudes into use. Without such comprehensive approaches, female condoms will likely remain underutilized despite their proven potential for empowering women and protecting against both unintended pregnancies and sexually transmitted infections.

### Implications for practice and public health

The findings of this study have important implications for nursing education, sexual and reproductive health practice, and public health policy. First, nursing curricula should integrate comprehensive female condom education that goes beyond theoretical knowledge to include practical demonstrations, hands-on practice with insertion, and role-playing scenarios for partner communication. Second, nursing training institutions should ensure consistent availability of female condoms at college health clinics and distribute samples to students for familiarization. This would address the access barrier and provide opportunities for students to practice before counselling patients.

Third, sexual and reproductive health education programs should adopt a couple-centered approach that engages male partners and addresses negotiation dynamics. Programs that build self-efficacy and communication skills have shown promise in improving female condom uptake. Furthermore, health policymakers should strengthen supply chain systems for female condoms and incorporate female condom promotion into national reproductive health campaigns. Social marketing strategies that normalize female condoms and challenge stigmatizing norms are essential.

### Limitation

Our study acknowledges the following limitations. The study was conducted in a single nursing training institution, which may limit the generalizability of findings to other settings or healthcare training programs. Data were self-reported, which may introduce social desirability bias, particularly regarding sexual activity and contraceptive use. The rare outcome of female condom utilization (7.7%) resulted in a relatively small number of positive events, which likely affected the stability of the logistic regression estimates, as evidenced by the wide confidence interval for marital status. Also, the attitude items assessed multiple dimensions of perceptions toward female condom use and were not based on a previously validated psychometric scale. Therefore, the attitude findings should be interpreted cautiously.

## Conclusion

While our study found a high positive attitude, actual utilization was low, as well as knowledge. This highlights that favourable perceptions alone may not translate into utilisation, particularly in the presence of limited knowledge and other barriers. The attitude-utilization gap highlights that favorable perceptions alone are insufficient for behavior change without addressing structural barriers, including access, practical skills training, and partner dynamics. Marital status significantly predicted utilization, further emphasizing the central role of interpersonal dynamics and negotiation power in contraceptive decision-making. Our findings suggest that favorable attitudes alone are insufficient for behavior change without a supportive system that enhances skills, confidence, and availability. Strengthening nursing education with comprehensive training on female condoms is essential to improve both personal uptake and professional advocacy. Integrating hands-on demonstrations of female condom insertion and use, enhanced availability of female condoms at college health facilities, incorporation of partner communication and negotiation skills training, and the development of peer education programs may help translate positive attitudes into sustained use. Addressing the identified gaps is therefore critical in achieving SDG target 3.7, which focuses on protecting the SRH of young women and also for advancing national efforts to reduce unintended pregnancies and STIs among young women in Ghana.

## Data Availability

The original contributions presented in the study are included in the article/Supplementary Material, further inquiries can be directed to the corresponding author.
