# Supplementary material for: Knowledge, attitudes, and practice gap in female condoms utilization among future health professionals. A cross-sectional study
Source: Front Reprod Health. 2026 Jun 18;8:1851756. doi: 10.3389/frph.2026.1851756 (PMC13323238; doi:10.3389/frph.2026.1851756)
Supplement: Supplementary file 1 [file Table1.docx]

**Section A: Socio-Demographic**

1. How old are you? …………….
2. Marital Status:
   - Never married
   - Married
   - Divorced
   - Separated
   - Widowed
3. Ethnicity:
   - Akan
   - Ewe
   - Ga-Dangme
   - Mole-Dagbani
   - Other (specify)………..
4. What is your religion?
   - Christianity
   - Islam
   - African Traditional
   - Other (specify)………
5. What is your academic level?
   - First year
   - Second year
   - Third year
6. Which program do you offer?
   - Registered Community Nursing (RCN)
   - Registered General Nursing (RGN)
   - Registered Nurse Assistant, Preventive (RNAP)
7. For the past seven months are you been sexually active?
   - Yes
   - No

**Section B: Knowledge of Female Condoms**

1. A female condom is designed to:
   - Prevent pregnancy only
   - Prevent STIs/HIV only
   - Prevent both pregnancy and STIs/HIV
   - Protect against cervical cancer
   - I don’t know
2. Which materials are used to make female condoms?
   - Latex
   - Polyurethane
   - Nitrile
   - Leather
   - I don’t know
3. Female condoms are most effective when:
   - Used with a male condom simultaneously
   - Used correctly and consistently every time
   - Doubled (using two at once)
   - All of the above
4. Where have you learned about female condoms? *(Select all that applies)*
   - College curriculum/lectures
   - Health workers/clinics
   - Friends/peers
   - Social media/radio/TV
   - Public health campaigns
   - Never learned about them
5. Female condoms can be inserted up to 8 hours before sex.
   - True
   - False

**Section C: Attitudes Toward Female Condoms**

|  | Strongly Disagree | Disagree | Neutral | Agree | Strongly Agree |
| --- | --- | --- | --- | --- | --- |
| 1. Cultural or religious norms make it difficult to use female condoms. |  |  |  |  |  |
| 1. Female condoms give women more control over their sexual health. |  |  |  |  |  |
| 1. I would feel embarrassed to suggest using a female condom to a partner |  |  |  |  |  |
| 1. Female condoms are a reliable method for preventing STIs and pregnancy. |  |  |  |  |  |
| 1. Nursing students should advocate for female condom use in their communities |  |  |  |  |  |
| 1. If available, how likely would you be to use a female condom? | Very unlikely | Unlikely | Neutral | Likely | Very likely |
|  |  |  |  |  |  |

**Section D: Utilization of female condom**

1. Have you ever used a female condom?

- Yes
- No *(If no, skip to Q22)*

1. If yes, when was the first time you used?

- 0 -3 months ago
- 4- 9 months ago
- 10 -12 month ago

1. If you have not used female condoms, why? *(Select all that applies)*
   - Never heard of them
   - Difficult to access
   - Partner refuses
   - Prefer other contraceptives
   - Uncomfortable to use
   - Other:……………
2. Where do you assessed it the last time you used it? *(Select all that applies)*
   - College clinic
   - Local pharmacy
   - Government health facility
   - Friends
   - Don’t know
3. Have you ever discussed female condoms with a healthcare provider?
   - Yes
   - No
4. What challenges do you face in using female condoms (Select all that apply)?

- **Difficulty accessing female condoms**
- **Partner refuses to use them.**
- **Cultural stigma**
- **Lack of awareness about how to use female condoms correctly.**
- **Discomfort or difficulty during insertion/use.**
- **Negative perceptions**
- **Prefer other contraceptive methods**
- Other:..…….
